# Advances and Opportunities of CRISPR/Cas Technology in Bioengineering Non-conventional Yeasts

**DOI:** 10.3389/fbioe.2021.765396

**Published:** 2021-10-11

**Authors:** Lu Shan, Zongjie Dai, Qinhong Wang

**Affiliations:** ^1^ Key Laboratory of Systems Microbial Biotechnology, Tianjin Institute of Industrial Biotechnology, Chinese Academy of Sciences, Tianjin, China; ^2^ National Center of Technology Innovation for Synthetic Biology, Tianjin, China

**Keywords:** CRISPR/cas, cell factories, microbial production, non-conventional yeast, genome engineering

## Abstract

Non-conventional yeasts have attracted a growing interest on account of their excellent characteristics. In recent years, the emerging of CRISPR/Cas technology has improved the efficiency and accuracy of genome editing. Utilizing the advantages of CRISPR/Cas in bioengineering of non-conventional yeasts, quite a few advancements have been made. Due to the diversity in their genetic background, the ways for building a functional CRISPR/Cas system of various species non-conventional yeasts were also species-specific. Herein, we have summarized the different strategies for optimizing CRISPR/Cas systems in different non-conventional yeasts and their biotechnological applications in the construction of cell factories. In addition, we have proposed some potential directions for broadening and improving the application of CRISPR/Cas technology in non-conventional yeasts.

## Introduction

Non-conventional yeasts have been considered as potential eukaryotic chassis for scientific research and industrial application. Owing to their outstanding natural characteristics. These advantageous attributes include thermotolerance, utilization of extensive carbon sources, and the capacity to produce high-titer proteins, lipids, or other commercial metabolites. For instance, *Scheffersomyces stipitis* is continually used in ethanol fermentation via lignocellulosic feedstock due to its inherent xylose metabolism (Agbogbo and Coward-Kelly, 2008). Methylotrophic yeasts like *Ogataea polymorpha*, *Pichia pastoris* and *Ogataea thermomethanolica*, possess an efficient ability to secrete heterologous protein and glycosylate, and were utilized commercially for producing a variety of proteins (Gellissen, 2000; Hartner and Glieder, 2006; Bredell et al., 2018). Oleaginous yeasts *Rhodotorula toruloides* and *Yarrowia lipolytica*, are capable of storing large amounts of cellular lipids from low-cost carbon sources (Castañeda et al., 2018; Chattopadhyay et al., 2021). The heat resistance of *Kluyveromyces marxianus* allows it to ferment at higher temperature, thus decreasing the probability of contamination (Marcišauskas et al., 2019). Furthermore, *Kluyveromyces lactis* finds extensive usage in lactose metabolism to secrete proteins (Spohner et al., 2016).

Genetic engineering is fundamental to study gene functions and control the expression of genes for producing specific compounds or otherwise regulating the gene expression when these yeasts are employed for scientific research or industrial applications. The efficient genome editing approaches and the corresponding tools are critical for rapid genome and metabolic engineering. The traditional gene manipulation tools such as *Cre-loxP* system can improve the genome editing efficiency to some extent, but these systems can only modify single locus in one step, and the marker recycling is time-consuming and left many scars in genome, which is not conducive to genomic stability (Xie et al., 2014). Moreover, the natural homologous recombination (HR) depends on a DNA break which occurs accidentally at the target locus (Raschmanová et al., 2018). In the last few decades, several new, better, and more accurate genetic tools have been developed to improve the efficiency of genome editing, such as zinc-finger nucleases (ZFNs) (Doyon et al., 2011) and transcription activator-like effector nucleases (TALENS) (Li et al., 2011). The targeting of specific DNA sequences by ZNFs and TALENs depends on the protein-DNA interaction, and then the DNA break is introduced by FokI. However, the construction of specific DNA binding proteins is still a laborious and time-consuming task.

**FIGURE 1:**
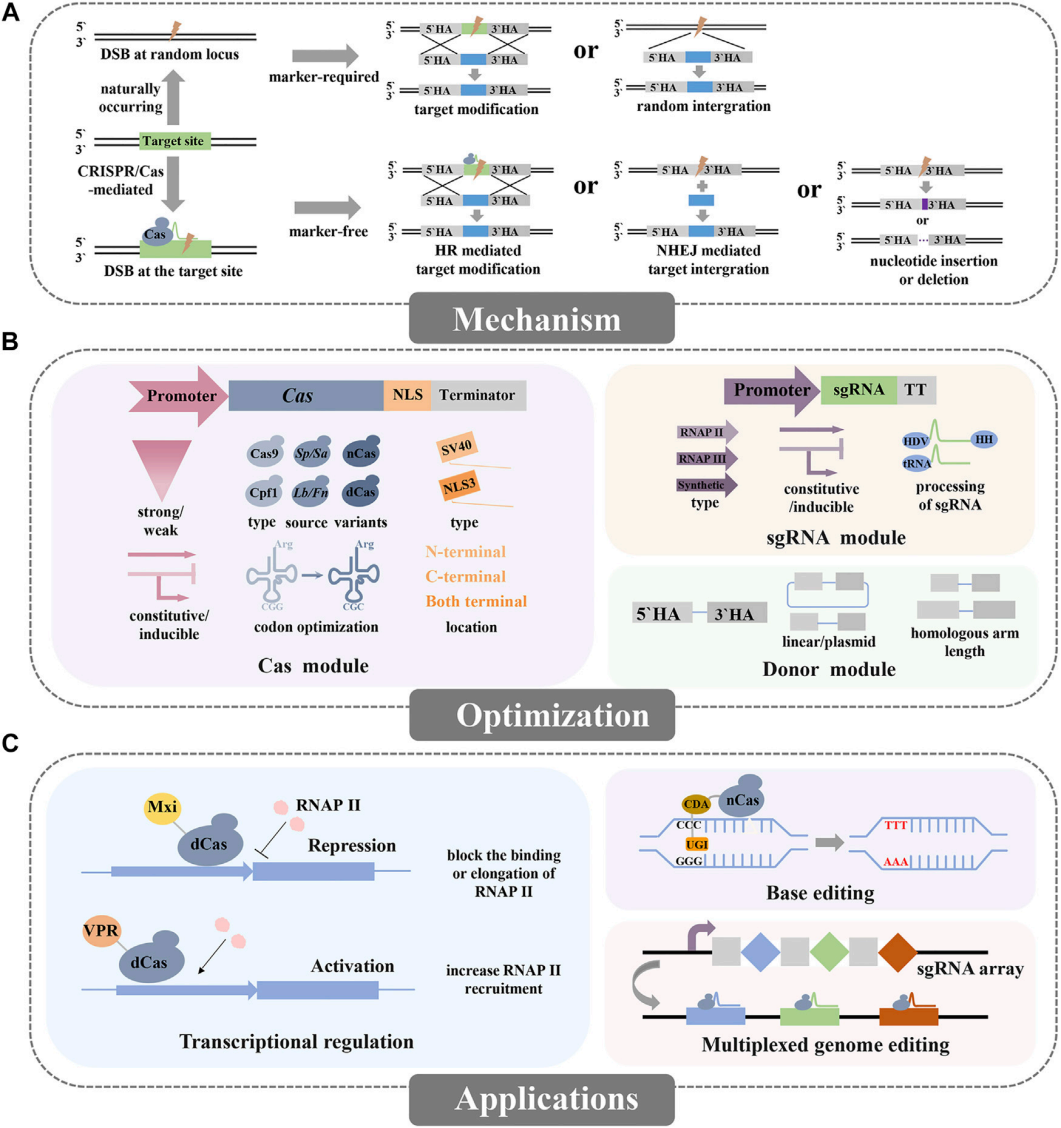
Overview of the mechanism, optimizations, and applications for CRISPR/Cas mediated genome editing in non-conventional yeasts. **(A)** Genome editing via naturally occurring and CRISPR/Cas-mediated DNA repair mechanisms. **(B)** The optimizations of the CRISPR/Cas system include the Cas module, sgRNA module, and donor module. **(C)** The applications of the CRISPR/Cas system mainly include the basic applications (knockin or knockout) and advanced applications (repression, activation, base editing or multiplexed genome editing).

Recently, the CRISPR/Cas system has revolutionized genome editing technology due to its efficiency, accuracy, and convenience (Hsu et al., 2014). This system essentially comprises a DNA endonuclease (e.g. Cas9 or Cpf1) which can bind to the target DNA sequence by the guidance of sgRNA, and then generating DNA double-strand breaks (DSB), and later the repair mechanism including HR, non-homologous end-joining (NHEJ), or microhomology-mediated end joining (MMEJ) is activated (Zetsche et al., 2015; Burstein et al., 2017) (Figure 1A). The CRISPR/Cas system relies on the DNA-RNA recognition for inducing precise DNA cleavage, which is marker-free and capable of simultaneous multi-loci editing, thus greatly accelerates the genome editing.

The genome-editing system mediated by CRISPR/Cas has already been widely employed in the genetic engineering of non-conventional yeasts and promoted the biotechnological development of these yeasts. Considering the increasing attention of non-conventional yeasts as the chassis for synthetic biology, we systematically summarized the optimization strategies for highly efficient adopting CRISPR/Cas systems in non-conventional yeasts and highlighted the advanced applications of this technology on functional genomics and constructing non-conventional yeast cell factories. Based on current achievements and challenges, we presented our perspectives on building more efficient and adaptable CRISPR/Cas derived system, which would provide new insights in further study CRISPR/Cas technology in non-conventional yeasts.

## Optimization of CRISPR/Cas System in Non-conventional Yeast

CRISPR/Cas system comprises two main components, namely Cas protein and sgRNA. To ensure efficient genome editing in non-conventional yeasts, species-specific optimizations of these two components are essentially required (Figure 1B).

### Optimization of sgRNA Expression Through Different Promoters

Sometimes the efficient expression of sgRNA comes out to be a challenge in non-conventional yeasts due to the lack of suitable promoters. The promoters used for sgRNA expression should have suitable strength and the sgRNAs require nuclear localization and both ends trimming. In non-conventional yeasts, the transcription of functional sgRNA is usually achieved by four types of promoters: 1) The RNA polymerase II (RNAP II) dependent promoters; 2) The RNA polymerase III (RNAP III) dependent promoters; 3) Synthetic hybrid promoters; 4) T7 polymerase dependent artificial promoter.

The RNAP II dependent promoters are usually responsible for producing mRNAs, so the ribozymes executing cleavage sequences are generally flanked on both ends of sgRNAs for modification and maturation. Hammer head ribozyme (HHR) and hepatitis delta virus (HDV) ribozyme are the commonly used elements, which have been employed in building CRISPR/Cas9 system in *S. cerevisiae* (Gao and Zhao, 2013), *P. pastoris* (Weninger et al., 2016), *O. thermomethanolica* (Phithakrotchanakoon et al., 2018), and *Y. lipolytica* (Gao et al., 2016). However, the genome editing efficiency was lower in comparison to RNAP III dependent promoters based CRISPR/Cas system. This may be presumably due to the extension of the sgRNA variable region from 20 bp to 69 bp because of the homologous arm of the 5′ hammerhead ribozyme (Gao and Zhao, 2013), another reason may be the sgRNAs transcribed by these promoters were exported from the nucleus into the cytosol for translation. Hence RNAP II dependent promoters have rarely been used in sgRNA expression. Interestingly, in the CRISPR/Cpf1 system of *Y. lipolytica*, the editing efficiency reached 93.3% ± 11.5% where sgRNA expression was controlled by RNAP II dependent promoter *TEFin* without ribozymes (Yang et al., 2020), which may be caused by the inherent capacity of Cpf1, thereby enabling to produce mature sgRNA from pre-sgRNA array (Fonfara et al., 2016).

RNAP III dependent promoters such as *SNR52*, *RPR1* have been considered as the most suitable candidates for the expression of sgRNA, because the RNAs transcribed by them remain in the nucleus. By far, the *SNR52* promoter is the most commonly employed promoter for sgRNAs expression, which allowed for gene editing in various non-conventional yeasts, including *S. stipites*, *K. marxianus*, *K. lactis*, *C. albicans and O. polymorpha.* The *RPR1* promoter was also employed in *P. kudriavzevii* and *I. orientalis*. In Table 1, we summarized the great and broad effect of these type of promoters in genetic engineering of non-conventional yeasts.

**TABLE 1:** Genetic editing applications of CRISPR/Cas in non-conventional yeasts.

Organism	Host strains	Expression cassette for cas	Promoter of sgRNA	Application	References
*Y. lipolytica*	PO1f	*P*_*UAS1B8-TFF (136)*_-*Sp_Cas9*-*T*_*CYC1*_	^a^ *SCR1’-tRNA* ^*Gly*^	Lycopene biosynthesis pathway (*crtB*, *crtE*, *crtI* and *Ggs1*) were integrated into different loci, resulting in an 8.6 folds increase in lycopene production over the wildtype strain	Schwartz et al. (2017b)
		(*Y. lipolytica* codon-optimized)			
—	PO1f	*P*_*UAS1B8-TFF (136)*_-*Sp_Cas9*-*T*_*CYC1*_	^a^ *SCR1’-tRNA* ^*Gly*^	A dual-cleavage strategy for gene excision and targeted integration	Gao et al. (2018)
		(*Y. lipolytica* codon-optimized)			
—	PO1f *ku70Δ*	*P*_*TFFin*_-*Sp_dCas9*-*T*_*XPR2*_ (*Y. lipolytica* codon optimized)	^a^ ^,^ ^b^ (*A1R1*)_*x2*_ *A3*	Combing of CRISPRi and a sensor of fatty acid to achieve negative autoregulation of the lipogenic pathway, the naringenin production was increased by 74.8% resultantly	Lv et al. (2020)
—	PO1f	*P*_*UAS1B8-TFF (136)*_-*Sp*_*dCas9*-*VPR*-*T*_*CYC1*_	^a^ *SCR1’-tRNA* ^*Gly*^	Two genes for cellobiose metabolism were activated through CRISPRa, enabling growth with cellobiose as the single carbon source	Schwartz et al. (2018)
		(*Y. lipolytica* codon-optimized)			
—	PO1g *ku70Δ*	*P*_*UAS1B8-TEF (136)*_ *-Sp_nCas9-pmCDA1-UGI-T*_*CYC1*_	^a^ *SCR1’-tRNA* ^*Gly*^	The C to T mutation enabled to introduce a stop codon to achieve the disruption of target gene. The efficiency of single and simultaneous double gene disruption was 94% and 31% *via* optimizing expression level (*P*_*TFFin*_ *-nCas9-pmCDA1-UGI-T*_*CYC1*_)	Bae et al. (2020)
		*P*_*TFF*_ *-Sp_nCas9-pmCDA1-UGI-T*_*CYC1*_			
		*P*_*EXP*_ *-Sp_nCas9-pmCDA1-UGI-T*_*CYC1*_			
		*P*_*TFFin*_ *-Sp_nCas9-pmCDA1-UGI-T*_*CYC1*_ (*Y. lipolytica* codon-optimized)			
*O. polymorpha*	CGMCC7.89	*P*_*TEF1*_ *-Sp_Cas9-T*_*ADH2*_ (*H. sapiens* codon optimized)	^c^ *SNR52*	A CRISPR–Cas9-assisted multiplex genome editing (CMGE) system was applied for multigenic editing include multiloci and multicopies. The efficiency of triple different genes knockout and knockin was 23.6% and 30.5% respectively. Multicopies GFP and resveratrol biosynthesis pathways were integrated into the rDNA site	Wang et al. (2018)
—	BY4330	*P*_*TEF1*_ *-Sp_Cas9-T*_*TEF1*_ (*H. sapiens* codon-optimized)	^a^ *SNR6-tRNA* ^*CUG*^	A tRNA-sgRNA fusion was developed for efficient genome editing, the efficiency of disrupting target genes (four genes) via indel mutations ranged from 17% to 71%	Gao et al. (2021)
*I. orientalis*	SD108	*P*_*TEF1a*_ *-Sp_iCas9-T*_*PGK1*_ (*iCas9*: *SpCas9* with two mutations D147Y and P411T; *H. sapiens* codon optimized) (Bao et al., 2015)	^c^ *tRNA* ^*Leu*^	A series of native and synthetic promoters for sgRNA expression were characterized. The highest efficiency was achieved by *RPR1’-tRNA* ^*Leu*^ promoter with the efficiency of single, double, and triplexed gene disruption was 97%, 90%, and 46.7%	Tran et al. (2019)
			^c^ *tRNA* ^*ser*^		
			^c^ *RPR1*		
			^c^ *5S rRNA*		
			^a^ *RPR1’-tRNA* ^*Leu*^		
			^a^ *5S rRNA-tRNA* ^*Leu*^		
*P. kudriavzevii*	YB4010	*P*_*TDH3*_ *-Sp_iCas9-T*_*TEF1a*_ (*H. sapiens* codon optimized)	^c^ *RPR1*	Deleting the downstream competing pathway and integrating the itaconic acid (IA) biosynthesis pathway into the genome. The production of IA was achieved at 1,232 mg/L in fed-batch fermentation	Sun et al. (2020)
*O. thermomethanolica*	TBRC656	*P*_*AOX*_ *-Sp_Cas9-T*_*AOX*_ (*H. sapiens* codon-optimized)	^b^ *AOX*	Integrative and episomal CRISPR systems were developed for gene disruption via indel mutations. Via the integrative system, three genes (*OtHAC1*, *OtMAL1* and *OtMAL2*) were disrupted with the efficiency of 63%, 97%, and 93%, individually. Utilizing the episomal system, the efficiency of one gene (*OtMAL1*) disruption was 92%	Phithakrotchanakoon et al. (2018)
*C. albicans*	SC5314	*P*_*ENO1*_ *-Ca_Cas9-T*_*ENO1*_ (*C. albicans* and *S. cerevisiae* codon-optimized)	^c^ *SNR52*	Four loci (*CDR1* and *CDR2*, two alleles each) were targeted by a single sgRNA via homozygous knockout, with an efficiency of 20%	Vyas et al. (2015)
*K. lactis*	ATCC 8585 *ku80Δ*	*P*_*FBA1*_ *-Sp_Cas9-T*_*CYC1*_ (*S. cerevisiae* codon-optimized)	^c^ *SNR52*	The muconic acid biosynthesis pathway (*AroF*, *AroB*, *AroD*, *AroZ*, *AroY* and *CATA*) was divided into three fragments targeting different loci. The efficiency of triple integrations was 2.1%	Horwitz et al. (2015)
*K. marxianus*	NBRC1777	*P*_*PDC1*_ *-Sp_nCas9-CDA-T*_*TDH3*_	^c^ *SNR52*	The Target-AID base editor was applied to introduce stop codon into *Nej1* and *Dnl4* genes through C-T mutagenesis. The efficiency of the mutant allele (*Nej1* and *Dnl4*) was 12.5%. Three DNA fragments were assembled and integrated with a 50 bp homology arm in NHEJ null mutants. The efficiency of integration was 100%	Nambu-Nishida et al. (2017)
		*P*_*PDC1*_ *-Sp_Cas9-T*_*TDH3*_ (*H. sapiens* codon optimized)			
—	CBS 6556	*P*_*TEF1*_ *-Sp_Cas9-T*_*CYC1*_ (*H. sapiens* codon-optimized)	^c^ *SNR52*	A variety of natural and synthetic RNAP III dependent promoters were designed for the expression of sgRNA. The CRISPR/Cas9 system was employed to disrupt the genes of ethyl acetate biosynthesis for gene functional characterization. The highest editing efficiency (66%) was provided by the *RPR1-tRNA* ^*Gly*^ promoter. The efficiency of genes disruption ranged from 10% to 67%	Löbs et al. (2017)
			^c^ *tRNA* ^*Gly*^		
			^a^ *SNR52-tRNA* ^*Gly*^		
			^a^ *SCR1-tRNA* ^*Gly*^		
			^a^ *RPR1-tRNA* ^*Gly*^		
—	CBS 6556	*P*_*TEF1*_ *-Sp_dCas9-T*_*CYC1*_ (*H. sapiens* codon-optimized)	^c^ *tRNA* ^*Gly*^	A multiplexed CRISPRi approach was used for regulating the central carbon flux to increase the production of ethyl acetate. The production of ethyl acetate is 3.8-fold higher than the natural capacity	Löbs et al. (2018)
*R. toruloides*	NP11	*P*_*GPD*_ *-Sa_Cas9-T*_*HSP*_ (*R. toruloides* codon optimized)	^c^ *U6b*	The CRISPR/Cas9 system was developed for gene disruption. The genes (*CRT1*, *CAR2*, *and CLYBL*) disruption efficiency via indel mutations was 66.7%, 75%, and 75%, respectively. The disruption efficiency of gene *CRT1* via HR was 8%	Jiao et al. (2019)
—	NP11	*P*_*GPD1*_ *-Sp_Cas9-T*_*NOS*_	^b^ *GPK*	An optimal CRISPR/Cas9 system was developed for multiplexed gene disruption. *PGK1* promoter for Cas9 and *5S-tRNA* ^*Gly*^ promoter for sgRNA are the best combination, the efficiency of duplexed gene disruption was 78%	Schultz et al. (2019)
		*P*_*FBA1*_ *-Sp_Cas9-T*_*NOS*_	^c^ *tRNA* ^*Gly*^		
		*P*_*PGI1*_ *-Sp_Cas9-T*_*NOS*_	^c^ *5S*		
		*P*_*PGK1*_ *-Sp_Cas9-T*_*NOS*_	^a^ *5S-tRNA* ^*Gly*^		
		*P*_*TEF1*_ *-Sp_Cas9-T*_*NOS*_ (*R. toruloides* codon-optimized)	^a^ ^,^ ^b^ *GPK-5S-tRNA* ^*Gly*^		
*S. stipitis*	Y-21448	*P*_*ENO1*_ *-Sp_Cas9-T*_*TEF1*_ (*S. stipitis* codon-optimized)	^c^ *SNR52*	The efficiency of gene disruption *via* indel mutations was 80%	Cao et al. (2017)
—	Y-21448	*P*_*ENO1*_ *-Sp_Cas9-T*_*TEF1*_	^c^ *SNR52*	Gene knockin via HR in NHEJ-deficient strain. The dCas9-Mxi1 was used to repress the expression of eGFP. The efficiency of gene knocking was 73–83%. The eGFP expression was repressed by 32–40% via targeting to different loci	Cao et al. (2018)
		*P*_*ENO1*_ *-Sp_dCas9-Mxi1-T*_*TEF1*_ (*S. stipitis* codon-optimized)			
*P. pastoris*	GS115	*P*_*HXT1*_ *-Sp_Cas9-T*_*DAS1*_ (*H. sapiens* codon optimized)	^b^ *HXT1*	High-efficiency sites were screened for multiloci gene integration. Three high-efficiency sites were screened, the efficiency of double-locus and triple-locus integration was 57.7–70% and 12.5–32.1%	Liu et al. (2019)
—	GS115	*P*_*GAP*_ *-Sp_Cas9-T*_*AOX1*_	^b^ *HTX1*	Episomal expression of sgRNA was used for CRISPR/Cas9, and the dCas9-based CRISPRi was introduced for gene interference. The editing efficiency of each targeted gene reached or exceeded 75%, and a precise sequence of *P*_*AOX1*_ which can control the transcription and translation of *AOX1* was obtained *via* CRISPRi	Hou et al. (2020)
		*P*_*GAP*_ *-Sp_dCas9-T*_*AOX1*_ (*H. sapiens* codon optimized)			

a Synthetic promoter.

b RNAP II dependent promoter.

c RNAP III dependent promoter.

Sp_Cas9: *Streptococcus* pyogenes Cas9, Sa_Cas9: *Staphylococcus aureus* Cas9, Ca_Cas9: non-canonical codon Cas9 for C. albicans.

The RNAP III binding sites of some RNAP III dependent promoters are located within their mature transcript, which may add additional nucleotides to the sgRNA, thereby preventing the maturity and release of sgRNA. The sgRNAs expressed by these promoters are thus fused with tRNA, then the tRNA is isolated through its internal maturation mechanism. In *P. pastoris*, the orthogonal tRNA-sgRNA cassettes were expressed by the *tRNA* promoter that enabled multiplexed genome integration of three genes (*gnt1*, *mns1*, and *mnn2*) involved in glycosylation (Dalvie et al., 2020). In *O. polymorpha*, an improved system with tRNA^Leu^-sgRNA fusion was constructed to enhance the sgRNA expression. The efficiencies of indel mutations were significantly improved to 17–71% in comparison to native RNAP III dependent promoter *OpSNR6* which resulted in less than 1% gene disruption (Gao et al., 2021). In *I. orientalis*, a series of native and synthetic promoters used for sgRNA expression were evaluated, and the synthetic *RPR1’-tRNA* ^*Leu*^ promoter was identified as the most effective promoter; the efficiency of single, double, and triple gene disruption was recorded as 97, 90, and 46.7% (Tran et al., 2019), respectively. A similar study was up taken in *Y. lipolytica* where synthetic promoters based on the RNAP III dependent promoters and tRNA^Gly^ were employed for the expression of sgRNA. The editing efficiency of *PEX10* reached up to 92% by the synthetic promoter *SCR1’-tRNA* ^*Gly*^, which enabled more than a 2-fold increase over the native *SNR52* promoter (Schwartz et al., 2016). In *K. marxianus*, three synthetic RNAP III dependent promoters, including *RPR1-tRNA* ^*Gly*^, *SCR1-tRNA* ^*Gly*^, and *SNR52-tRNA* ^*Gly*^ promoter, were applied to optimize the expression of sgRNA. The highest editing efficiency observed in this case was 66% which was achieved by the *RPR1-tRNA* ^*Gly*^ promoter (Löbs et al., 2017). Similarly, in the CRISPR/Cpf1 system, synthetic *SCR1’-tRNA* ^*Gly*^ promoter and native the RNAP III dependent promoter *5sRNA* were tested for their potential to enhance sgRNA expression in *Y. lipolytica*. The highest efficiency was achieved in the case of *SCR1′-tRNA* ^*Gly*^ promoter; the editing efficiency of *CAN1 via* indels mutation was found to be as high as 86.6% ± 5.7% (Yang et al., 2020).

However, finding a suitable RNAP III dependent promoter in some hosts is still a challenge. In a recent study, an artificially constructed promoter based on the T7 expression system was successfully used for sgRNA expression in some yeasts. An optimized T7 polymerase mutant (P266L) fused with an SV40 nuclear localization sequence (NLS) was developed to ensure a functional T7 promoter for the expression of sgRNA. This system was widely applied in *Y. lipolytica*, *K. lactis*, and *S. cerevisiae* and its editing efficiency was more than 60% (Morse et al., 2018). The optimized T7 expression system from bacteria provides an alternative tool for hosts with no suitable promoters for sgRNA expression. Overall, these innovative approaches of sgRNA expression have great potential for enhancing genome editing in non-conventional yeasts.

### Strategies for Optimal Cas Protein Expression

Nuclear localization, codon optimization, and promoter screening (strong/weak or constitutive/inducible) were often adapted for optimizing the expression of Cas protein. In eukaryotic chassis, the Cas protein should be localized in the nucleus to generate DSB, the most common strategy being the fusion of an NLS with the Cas to achieve nuclear targeting. The most typical strategy involves the fusion of an SV40 NLS to the N- or C-terminal of Cas (Schwartz et al., 2016; Yang et al., 2020), or even both C- and N-terminal (Cao et al., 2017; Tran et al., 2019; Hou et al., 2020). It has been reported that in *R. toruloides*, an SV40 NLS fused to the C-terminal of Cas9 is insufficient to achieve its nuclear targeting. Therefore, NLS3 (an endogenous NLS) was appended to the C-terminus of the Cas9 to ensure its import to the nucleus (Schultz et al., 2019).

Codon optimization of the *Cas* gene can affect the functionality of the CRISPR/Cas system, however, in terms of the efficiency of genome editing, it does not seem necessary in some non-conventional yeasts. The *Homo sapiens* codon-optimized *Cas9* has been used for genetic editing in *P. pastoris* (Weninger et al., 2016) and *Y. lipolytica* (Gao et al., 2016). *ScCas9* (*S. cerevisiae* codon-optimized) has been used in *K. lactis* (Horwitz et al., 2015) and *C. glabrata* (Enkler et al., 2016). Both *C. glabrata* and *S. cerevisiae* codon-optimized sequences of *Cas9* enabled the genetic editing in *C. glabrata* (Enkler et al., 2016) and both *H. sapiens* and *Y. lipolytica* codon-optimized sequences are functional in *Y. lipolytica* (Gao et al., 2016; Schwartz et al., 2016)*.* In contrast, the codon optimization of *Cas9* seriously influenced the efficiency of editing in *P. pastoris* (Weninger et al., 2016) and must be required for the non-canonical codon assignment yeast *C. albicans* (Vyas et al., 2015).

In non-conventional yeasts, the promoters used for *Cas* expression commonly focus on strong and constitutive promoters, such as *P*_*TEFin*_ *or P*_*TEF*_ in *Y. lipolytica* (Holkenbrink et al., 2018; Bae et al., 2020), *P*_*ENO1*_ in *C. albicans* (Vyas et al., 2015), *P*_*FBA1*_ in *K. lactis* (Horwitz et al., 2015), and *P*_*ScTEF1*_ in *C. glabrata* (Enkler et al., 2016). In *O. thermomethanolica*, the expression of Cas9 was controlled by an inducible promoter *P*_*AOX*_. The high expression of Cas9, when triggered by methanol, has a detrimental influence on its fitness. This negative effect was ameliorated when the expression of Cas9 was induced by glycerol instead (Schwartz et al., 2017a). However, the inducible promoter tested in *P. pastoris* was not successful (Schwartz et al., 2018). In *C. glabrata*, the choice of the promoter may influence the type of mutation, a single base pair or larger insertions were observed when different promoters were used to express *Cas9* (Enkler et al., 2016)*.* In *Y. lipolytica*, different promoters were screened for *nCas9-pmCDA1-UGI* expression, and the highest efficiency was achieved by *TFFin* promoter (Ramesh et al., 2020). These findings show that an appropriate level of *Cas9* expression is beneficial to the strain’s resilience and genome editing efficiency. As a result, species-specific improvements of the CRISPR system are required for it to function properly.

Except for the expression of sgRNA and Cas, the targeted gene loci and the sequence of sgRNA also have a significant impact on the genome-editing efficiency. In addition, if the DSB must be repaired by HR, a donor DNA must be given. The type (linear or plasmid) and homologous arm length of donor DNA also impact HR effectiveness.

## Advanced CRISPR/Cas Technology in Non-conventional Yeasts

Besides the exploitation of the basic function of the CRISPR/Cas system like gene deletion or integration, some advanced CRISPR/Cas technologies have been applied in non-conventional yeasts such as the regulation of transcription, base editing and homology-independent gene integration (Figure 1C).

### CRISPR Interference and CRISPR Activation

Metabolic engineering and functional genomics both benefit from targeted gene transcriptional regulation. CRISPR activation (CRISPRa) and CRISPR interference (CRISPRi) have been constructed to enhance or weaken the expression of the target gene in non-conventional yeasts. These technologies are based on inactive Cas (dCas), which still preserving the capacity to target and bind to particular DNA sequences.

CRISPRi is a simple but useful tool that can down-regulate the expression level of the target gene. By binding to region of the promoter, dCas could sterically block the binding or elongation of the RNA polymerase, resulting in transcription repression. The effect of repression can be further strengthened by fusing a repressor domain like Mxi1 to dCas. For example, a CRISPRi system based on dCas9 was developed in *P. Pastoris*, and through this system, a more precise sequence of *P*_*AOX*1_ was obtained which can control the transcription and translation of *AOX1* (Hou et al., 2020). In *Y. lipolytica*, the CRISPRi system was also developed to repress NHEJ. By multiplexed targeting to *ku70* and *ku80*, the HR efficiency, in this case, is as high as 90% compared to the *ku70* deficient strain (Schwartz et al., 2017a). In *K. marxianus*, a multiple CRISPRi system was developed for redirecting carbon flux of the central metabolic pathway towards ethyl acetate production, causing an improved ethyl acetate titer by 3.8-fold (Löbs et al., 2018). Alternatively, the selection of sgRNA has a great influence on the repression efficiency of CRISPRi. Zhang *et al.* designed a multiplexed sgRNA targeting strategy in *Y. lipolytica*. Through simultaneous targeting to *gfp* gene with three different sgRNAs, the repression efficiency reached 92% and 85% with dCas9 and dCpf1 respectively. Furthermore, the efficient repression of three target genes (*vioA*, *vioB*, and *vioE*) in protodeoxy-violaceinic acid (PVA) synthetic pathway was also realized in one step by this strategy, the content of PVA was reduced by 61% and 75% with dCpf1 and dCas9 separately compared with their corresponding control strains (Zhang et al., 2018).

In general, the fusion of transcriptional activators like VP64 and VPR with dCas9 causes gene upregulation by increasing RNA polymerases recruitment. In *Y. lipolytica*, a dCas9-VPR fusion was used to activate β-glucosidases that allow its growth with cellobiose as the single carbon source (Schwartz et al., 2018). Truncating sgRNA could inhibit the nuclease activity of Cas protein, but not influence the targeting effect. On this basis, a Cpf1-VPR fusion with truncated sgRNA (16 bp) increased hrGFP expression by 10-fold in *Y. lipolytica* (Ramesh et al., 2020).

In addition to the on/off states of gene expression, controlling the gene expression at a suitable level allows for the creation of the desired phenotype. Graded gene expression strengths were obtained by altering the sgRNA target site in the promoter region using CRISPRi or CRISPRa. It resulted in a dynamic gene expression range from zero to several 10-fold improvement, allowing for fine-tuning of metabolic pathway expression and optimization of optimal phenotypes. The balance between cell growth and products biosynthesis is the major issue to be addressed in construction cell factories. CRISPR/Cas-mediated multiple genes synchronized regulation maybe a powerful tool to build a highly efficient non-conventional yeast cell factories.

### Base Editing

Base editing is a valuable tool with a lot of potential in genetic editing. By fusing deaminase with the nCas or dCas proteins, these fusion proteins may directly create precise point mutations in genomic DNA. A Target-AID (target activation-induced cytidine deaminase) base editor based on the nCas9-pmCDA1 fusion protein was created to execute the conversion of C to T in human cells and *S. cerevisiae*, which was tested by inserting a nonsense mutation into the coding sequence.

In non-conventional yeasts, the Target-AID base editor was employed to realize gene disruption in *Y. lipolytica* (Bae et al., 2020). Similarly in *K. marxianus*, this base editor was also constructed to disrupt *Nej1* or *Dnl4* which was involved in NHEJ to enhance the proportions of HR-mediated integration (Nambu-Nishida et al., 2017). Apart from gene disruption, this base editor was further employed for situ mutagenesis, thus enabling it to obtain the desired phenotype. Recently, the general transcription factor gene *SPT15* in *S. cerevisiae* was mutated by Target-AID base editor to enhance the stress tolerances (Liu et al., 2021). Furthermore, this strategy has also been applied in mammals (Ma et al., 2016) and plant (Li et al., 2020) but has not been widely reported in non-conventional yeasts. Though the overall usage of base editing is less in comparison to conventional CRISPR/Cas genetic editing, it has the potential to complement genetic editing tools because of its procedures being donor-free and DSB-free.

### Homology-independent Targeted Genome Editing

Constructing homologous arms for homology-dependent genome editing is laborious and time-consuming. In most non-conventional yeasts such as *Y. lipolytica*, *S. stipites*, *R. toruloides*, *P. pastoris*, and *K. marxianus*, NHEJ is the dominant repair pathway of DSB. A recently study took advantage of this inherent property and constructed a CRISPR/Cas9-mediated homology-independent gene integration tool in *Y. lipolytica*. The targeted gene integration rate was up to 55% by optimizing the cleavage efficiency of Cas9, manipulating repair fidelity of NHEJ, cell cycle and integration sites. By using this tool, iterative integration of canthaxanthin biosynthesis pathway including four genes (*GGS1*, *carB*, *carRP* and *CrtW*) was achieved (Cui et al., 2021). It is worth noting that integration of an 8,417 bp fragment composed of *GGS1*, *carB*, and *carRP* into genome by one step may still be a challenge for the HR dependent targeted genome integration, indicating this tool paves a new avenue to realize the accurate and efficient targeted genome integration in some non-conventional yeasts.

## CRISPR/Cas technology in functional genomics and cell factory construction of non-conventional yeasts

Relative to the conventional yeast *S. cerevisiae*, the genome annotations for non-conventional yeast are not particularly thorough. In *Y. lipolytica*, CRISPR/Cas9 has been applied to research functional genomics through the construction of a sgRNA library that covers the whole genome and targeting 7,845 coding sequences (CDS). A total of 1,377 CDSs were identified as necessary CDSs by employing this approach. This sgRNA library facilitates the screening of growth and non-growth related phenotypes, such as canavanine resistance (Schwartz et al., 2019). In *P. pastoris*, one to three nucleotides have been precisely inserted or deleted at the S215 of the methanol expression regulator Mxr1, and the S215 also has been mutated to A215 through a single base replacement. The frameshift mutation of *Mxr1* resulted in almost no transcription of its target genes *DAS1*, *DAS2* and *AOX1*, with *AOX2* transcription, decreased by 40%. For the Mxr1^S215A^, the transcription of these four targeted genes was decreased by nearly 60% (Hou et al., 2020). In *K. marxianus*, the CRISPR/Cas9 system was developed to characterize functional genes within the ethanol and ethyl acetate biosynthesis pathway by disrupting the genes involved, thereby demonstrating that *ADH7* (alcohol dehydrogenase) played a major role as an alternative pathway for the biosynthesis of ethyl acetate (Löbs et al., 2017). These researches demonstrated that CRISPR/Cas was a valuable method for determining gene function and identifying candidate genes.

The creation and application of strains often require repetitive design-build-test cycles, the CRISPR/Cas method can speed up this process because it enables marker-free gene editing. Wang *et al.* used CRISPR/Cas9 to engineer *O. polymorpha* to produce resveratrol. By targeting the rDNA site, 10 copies of resveratrol biosynthetic pathway (three genes, composed of *TAL*, *4CL* and *STS*) were integrated into the genome, resulting in higher resveratrol production, which reached as high as 97.23 ± 4.84 mg/L, representing a 20-fold increase compared with single-copy integration (Wang et al., 2018). In *Y. lipolytica*, CRISPR/Cas9 was used by *Schwartz et al.* to screen integration loci that not only allow for high expression of the integrated gene but also have no detrimental impact on cell resilience after the gene has been integrated. Five of the 17 loci were found, and four genes (*crtB*, *crtE*, *crtI* and *Ggs1*) involved in the lycopene production pathway were each integrated into a separate locus enabling an 8.6 folds increase in lycopene production in comparison to the wildtype strain (Schwartz et al., 2017b). In *P. kudriavzevii*, the CRISPR/Cas9 was adapted to deleting the downstream competing pathway and then integrating the itaconic acid (IA) biosynthesis pathway into the genome. The production of IA achieved afterward was 1,232 mg/L in fed-batch fermentation (Sun et al., 2020). Overall, CRISPR/Cas technology is a powerful tool in bioengineering these traditional hardly genetically engineered yeasts for basic research or industrial applications (Table 1).

## Discussion

Despite the numerous successful applications of CRISPR/Cas technology in different non-conventional yeasts, there are still many obstacles to overcome and CRISPR/Cas based techniques to develop in non-conventional yeasts.

In most non-conventional yeasts, NHEJ weakens their precise genome engineering. To increase the HR efficiency, the most common strategy is to knock out or inhibit NHEJ-related genes such as *ku70/80*, *Nej1*, and *Dnl4*, which have proven effective in *Y. lipolytica* (Kretzschmar et al., 2013), *K. marxianus* (Nambu-Nishida et al., 2017)*.* However, many studies reported that the robustness of NHEJ-deficient cells is poor in comparison to NHEJ-proficient cells (Kretzschmar et al., 2013). Alternatively, some studies show that the HR efficiency can be enhanced by overexpressing HR-associated genes like *Rad51/52* and *Sae2*. Expression of the codon-optimized *ScRad52* demonstrated an obvious improvement in the HR efficiency of *Y. lipolytica* (Ji et al., 2020). The combined expression of *ScRad51*, *ScRad52*, and *ScSae2* significantly improved the HR rate of *O. polymorpha* (Gao et al., 2021). On the contrary, *ScRad51/Rad52* expressed in *P. pastoris* (Cai et al., 2021) and *S. stipitis* (Cao et al., 2018) had no distinct improvement in HR efficiency, but overexpression of endogenous *Rad51* and *Rad52* resulted in higher HR activity of *P. pastoris* (Cai et al., 2021). Presumably, the mechanism of DSB repair is mysterious and complex, the choice of proper genes and the expression strength of these genes are important to increase HR efficiency, which is essential for efficient operation of the multiple genes simultaneously in non-conventional yeasts. Alternatively, CRISPR/Cas9-mediated homology-independent targeted gene integration maybe a potential tool for precise genome engineering.

In addition to the current commonly used Cas9 and Cpf1, other Cas proteins have gradually started receiving attention. Recently, Walton *et al.* developed a near-PAMless SpCas9 variant (SpRY), which can target almost all PAMs (Walton et al., 2020). For Cpf1, two AsCpf1 variants were developed, which recognize TYCV and TATV PAMs, respectively (Gao et al., 2017). Cas13a previously known as C2c2 can induce precise cleavage of RNA, therefore, it can perform RNA interference without DNA damage (Cox et al., 2017). Furthermore, gene editing at the RNA level is reversible and changeable, it is a viable method for developing dynamic regulatory elements to control the level and timing of Cas13a expression. Cas14, which has only 400 to 700 amino acids, may target ssDNA and induce cleavage without requiring a specific sequence (Harrington et al., 2018). Up to now, the applications of Cas9, Cpf1 variants, Cas13a, and Cas14 are mainly focused on nucleic acid testing (Gootenberg et al., 2017; Ge et al., 2021) or mammalian (Gao et al., 2017; Walton et al., 2020) and plant cells (Abudayyeh et al., 2017). Based on their advantages, they will have promising potentials to be developed in non-conventional yeasts.

The use of the CRISPR/Cas system in conjunction with other methods or effectors has considerably increased the total capability of cellular engineering. In the realm of synthetic biology, the combination of dynamic regulation with CRISPR is highly efficient in improving biological processes. Recently, the muconic acid production in *E. coli* was increased by 1.3-fold through using an optogenetic CRISPRi system (Wu et al., 2020). Similar to *Y. lipolytica*, by the combination of a fatty acid biosensor and CRISPRi, the production of naringenin was increased by 74.8% (Lv et al., 2020). On the other hand, the target nucleotide diversification was performed by the fusion of nCas9 and error-prone DNA polymerase in *E. coli* (Halperin et al., 2018) and *S. cerevisiae* (Tou et al., 2020) respectively. Although the base editing accomplished by Cas-deaminases fusion has been widely investigated, it has only been reported in few non-conventional yeasts. The introduction of these systems into non-conventional yeasts would be a huge help in fine-tuning gene expression or global genome engineering, both of which are important techniques for building highly efficient cell factories or scientific research.
